# Ligand fitting with *CCP*4

**DOI:** 10.1107/S2059798316020143

**Published:** 2017-02-01

**Authors:** Robert A. Nicholls

**Affiliations:** a Structural Studies, MRC Laboratory of Molecular Biology, Francis Crick Avenue, Cambridge CB2 0QH, England

**Keywords:** *CCP*4, *Coot*, ligand fitting, model building

## Abstract

Crystal structures of protein–ligand complexes are often used to infer biology and inform structure-based drug discovery. Hence, it is important to build accurate, reliable models of ligands that give confidence in the interpretation of the respective protein–ligand complex. This paper discusses key stages in the ligand-fitting process, including ligand binding-site identification, ligand description and conformer generation, ligand fitting, refinement and subsequent validation. The *CCP*4 suite contains a number of software tools that facilitate this task: *AceDRG* for the creation of ligand descriptions and conformers, *Lidia* and *JLigand* for two-dimensional and three-dimensional ligand editing and visual analysis, *Coot* for density interpretation, ligand fitting, analysis and validation, and *REFMAC*5 for macromolecular refinement. In addition to recent advancements in automatic carbohydrate building in *Coot* (*LO*/*Carb*) and ligand-validation tools (*FLEV*), the release of the *CCP*4*i*2 GUI provides an integrated solution that streamlines the ligand-fitting workflow, seamlessly passing results from one program to the next. The ligand-fitting process is illustrated using instructive practical examples, including problematic cases such as post-translational modifications, highlighting the need for careful analysis and rigorous validation.

## Abstract 2

The process of ligand fitting with *CCP*4 is reviewed, including identifying ligand density in the map, ligand fitting, refinement and subsequent validation. Recent developments are discussed, and are illustrated using instructive examples demonstrating practical application.

## 1. Introduction   

Macromolecular crystallography is a useful technique for determining how ligands interact with proteins. Following structure determination, crystal structures of protein–ligand complexes are often used in structure-based drug design, calculation of interaction energies and protein-induced strain, and to make other biological inferences. In addition to being used by the original scientists who determined the structure, models of crystal structures deposited in the Protein Data Bank (PDB; Berman *et al.*, 2002 ▸) are also used by other scientists who analyse the deposited models, as well as in more general studies involving PDB data mining and analysis. Conclusions made using modelled crystal structures of protein–ligand complexes can be highly sensitive to model quality and errors. Indeed, small changes in atomic positions may have a substantial impact on perceived chemical interactions, potentially leading to different results in subsequent analyses. Consequently, it is important that crystal structures are as accurate as possible and sufficiently reliable to give a definitive answer as to the binding mode of the ligand.

The poor quality of many ligands in the PDB is concerning, as has been recognized in the literature in recent years (see, for example, Cooper *et al.*, 2011 ▸; Liebeschuetz *et al.*, 2012 ▸; Malde & Mark, 2015 ▸; Pozharski *et al.*, 2013 ▸; Reynolds, 2014 ▸; Weichenberger *et al.*, 2013 ▸). Indeed, it has been acknowledged that the quality of ligands in macromolecular models deposited in the PDB has been lacking, with many ligands being fitted incorrectly and/or with poor geometry. The focus on the development of improved software tools and new features for ligand modelling and analysis (specifically for ligand description, fitting, analysis and validation) will hopefully prevent so many errors in the future.

Problems encountered in ligand fitting are often resolution-dependent. At high resolution (>2 Å) the electron density for a ligand should clearly show the binding mode of the ligand. At lower resolutions (<3 Å) the interpretation of electron-density maps can become questionable. Questions asked may include whether or not a given blob of density relates to a particular ligand, the degree of confidence in a particular pose of that ligand, and whether more than one pose is consistent with the ‘blob’ of density in the map (a pose is a candidate binding mode). At medium resolution (2–3 Å), while the binding mode of the ligand is often clear, finer details such as the water structure around the ligand may not be clear in the electron-density maps.

In this article, we consider how the ligand-fitting process can be efficiently achieved using the modern *CCP4* v.7.0 suite (Winn *et al.*, 2011 ▸) from within the *CCP*4*i*2 environment. Specifically, we focus on the creation of ligand descriptions and conformers with *AceDRG* (Long *et al.*, 2017 ▸) and *RDKit* (Landrum, 2006 ▸), ligand editing and visualization with *JLigand* (Lebedev *et al.*, 2012 ▸) and *Lidia* (Emsley, 2017 ▸), ligand building and fitting with *Coot* (Emsley & Cowtan, 2004 ▸; Emsley *et al.*, 2010 ▸; Emsley, 2017 ▸) and refinement with *REFMAC*5 (Murshudov *et al.*, 1997 ▸, 2011 ▸). The *CCP*4*i*2 GUI, which manages projects, allowing sequences of tasks to be performed, holds all of this together; the results from one task/program are seamlessly used as input to the next without requiring the user to micromanage input/output files. It should be noted that the *CCP*4 suite also contains other software tools to aid such tasks that are described elsewhere and are not discussed here, including some that deserve a special mention: *ARP*/*wARP* (Beshnova *et al.*, 2017 ▸; Langer *et al.*, 2008 ▸) for automated model building, *Privateer* (Agirre, Iglesias-Fernández *et al.*, 2015 ▸) for automated detection, building and validation of carbohydrates, *CCP*4*mg* (McNicholas *et al.*, 2011 ▸) for visualization, analysis and production of quality graphics, and *PanDDA* (Pearce *et al.*, 2016 ▸) for ligand detection in high-throughput crystallography and drug discovery. Additionally, note that *Coot* is also distributed as part of other crystallo­graphic suites, such as *PHENIX* (Adams *et al.*, 2010 ▸), which contain equivalent tools to perform the tasks described in this article.

A brief overview of the ligand-fitting process is given below, before discussing each of the stages in more detail. Particular focus is given to the application of features implemented in *Coot* and integration within *CCP*4*i*2. Some of the problems encountered and issues that should be contemplated during ligand fitting are discussed.

## 2. Overview of the ligand-fitting process   

Typically, ligand fitting begins after all macromolecules have been built and refined. The first step is to identify any blobs of unmodelled density, which may correspond to ligand-binding sites. In some cases it may be clear that a blob corresponds to a particular ligand, but in other cases it may be less obvious (*e.g.* owing to problems encountered during co-purification; Fischer, Hopkins *et al.*, 2015 ▸). It may be useful to compile a list of potential compounds (buffer components *etc.*) that could reasonably correspond to the blobs, and attempt to fit each of them before deciding which is the most likely.

In order to fit a ligand it is necessary to have a set of starting coordinates and restraints: *i.e.* the relative positions of atoms in the molecule along with information regarding their connectivity and other energetic and chemical properties (bond distances, angles *etc.*). For more information on how restraint dictionaries are generated and used during the refinement of protein–ligand complexes, see below and Steiner & Tucker (2017 ▸).

Following the generation of starting coordinates, the initial structure can then be placed into the density and oriented so as to optimally coincide with the identified blob. Manual or automatic real-space refinement would then be performed in order to optimize the fit of the ligand to density. Ideally, the result is a modelled ligand that reasonably agrees with the electron density. Full-model refinement is then performed, before subsequent validation, analysis and visualization.

The steps in a typical ligand-fitting process using *CCP*4 programs are described below.

(i) Identification of ligand electron density (§[Sec sec3]3).
(ii) Ligand description and generation of conformers (§[Sec sec4]4).
(iii) Ligand fitting with *Coot* (§[Sec sec5]5).
(iv) Finding the ligand position and an initial pose (§[Sec sec5.1]5.1).
(v) Conformer generation (§[Sec sec5.2]5.2).
(vi) Post-translational modifications (§[Sec sec5.3]5.3).
(vii) Jiggle Fit (§[Sec sec5.4]5.4).
(viii) Carbohydrate building (§[Sec sec5.5]5.5).
(ix) Full-model refinement, validation, analysis and visual­ization (§[Sec sec6]6).

Subsequent sections will discuss various aspects of this procedure in more detail.

## 3. Identification of ligand electron density   

The first question to be answered is: is there any electron density in the map that corresponds to the ligand of interest? The answer to this question is quite often no, in which case further crystallization experiments may need to be undertaken (Mueller, 2017 ▸; Hassell *et al.*, 2007 ▸). Ligands are typically built after the rest of the model has been built and refined as well as reasonably possible (except perhaps for waters); this is usually assessed by considering the convergence of *R* factors, reduction of *R*_free_, satisfaction of geometry/chemical validation and inspection of electron-density maps. At this point, it is hoped that the ligands, if present, will be clearly visible in the difference density (*mF*_o_ − *DF*_c_) map (the difference map can readily be searched in *Coot* using the ‘Difference Map Peak’ tool found in the ‘Validate’ menu). If there is convincing electron density in the map that could correspond to the ligand, then one can proceed with fitting. It should be noted that crystallization and data-collection conditions, such as temperature, can have an effect on such factors and thus on the potential ability to model a ligand (Fischer, Shoichet *et al.*, 2015 ▸).

Commonly encountered problems include flexible ligands with only partial visibility, partial occupancies and, at higher resolutions, multiple conformations. At lower resolutions it can often be a challenge simply to identify which ligand/molecule corresponds to a given blob in the density, especially if a cocktail of compounds were present upon crystallization. Note that the presence of heavy atoms can help with ligand identification and placement, as can a series of related ligands with different chemical ‘side chains’.

## 4. Ligand description and conformer generation   

The *CCP*4/*REFMAC* monomer library (Vagin *et al.*, 2004 ▸) comprises pre-computed descriptions for many common ligands (such as peptides). The restraints for these ligands are already distributed as part of the *CCP*4 suite, and are automatically used during fitting and refinement without any manual intervention required.

If a ligand’s pre-assigned three-letter code is known (*e.g.* ATP for adenosine triphosphate) then ligand generation can be performed with ease: the ‘Get Monomer’ tool in *Coot* can be used to import the corresponding coordinates and restraints from the monomer library. Coordinates and restraints corresponding to ligands that are not present in the monomer library can sometimes be found online, for example from DrugBank (Law *et al.*, 2014 ▸).

**Figure 1:**
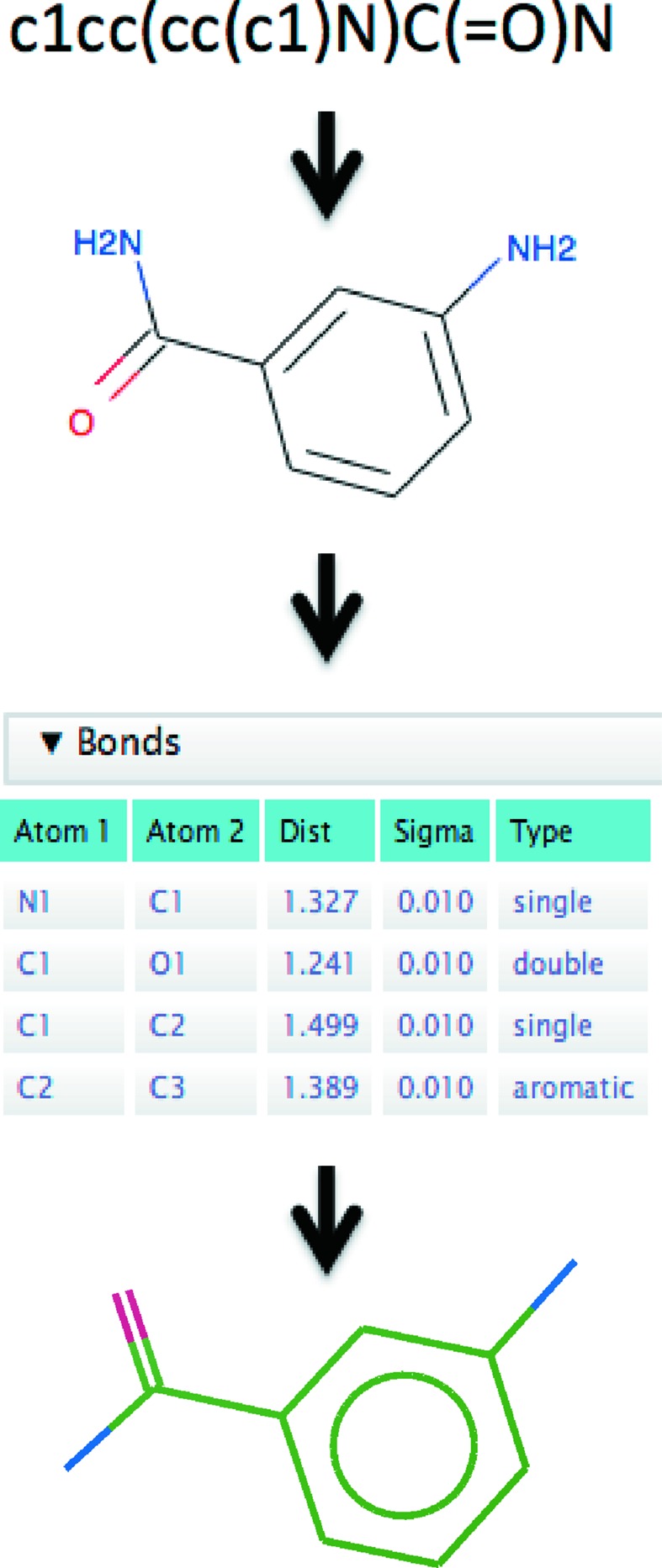
The process involved in generating a ligand description and conformer generation using *CCP*4/*AceDRG*. From an input SMILES string, the first step is to create a graphical representation of the ligand using *AceDRG*/*RDKit*, which encodes basic chemical properties and interactions. This can be depicted in two dimensions and edited using *Lidia*. A full ligand description is generated, with reference to prior knowledge specifying details of the restraints for bond lengths, angles, torsions *etc.*, using *AceDRG*. Using these restraints, *RDKit* generates an initial three-dimensional conformer before *REFMAC*5 is executed to optimize the coordinates.

If a full pre-computed ligand description is unavailable for a particular ligand, it is sufficient to start from a simple chemical description of the molecule that minimally contains information about the atomic elements and their connectivity. Such information can be found online or created manually using a ligand editor. From this starting point, a full ligand description can be generated in the form of restraints. These restraints can then be used to perform conformer generation in order to obtain a set of chemically reasonable initial atomic coordinates (see Fig. 1 ▸).

One tool for creating ligand descriptions and performing conformer generation is *AceDRG* (Long *et al.*, 2017 ▸), which was recently released as part of version 7.0 of the *CCP*4 suite. *AceDRG* takes a simple chemical description of a ligand (*e.g.* a SMILES string; Weininger, 1988 ▸) and uses it to generate a full ligand description (a CIF file) with reference to a database of prior knowledge. *AceDRG* then generates a starting conformer using *RDKit* (Landrum, 2006 ▸) to obtain the initial coordinates, before using *REFMAC*5 (Murshudov *et al.*, 1997 ▸; Murshudov *et al.*, 2011 ▸) to further optimize these coordinates, resulting in an output PDB file. Note that there is no reference to the density; at this stage the purpose is to obtain a ‘low-energy’ conformer according to the ligand description (*i.e.* the restraints), not to fit it into the density. Consequently, the resultant model may or may not adopt the same conformation as that of the true structure in the crystal. It should be noted that there are a variety of other tools available for dictionary and conformer generation, including *AFITT* (Wlodek *et al.*, 2006 ▸), *Corina* (Gasteiger *et al.*, 1990 ▸), *grade* (Smart *et al.*, 2011 ▸), *LIBCHECK* (Vagin *et al.*, 2004 ▸), *Mogul* (Bruno *et al.*, 2004 ▸), *phenix.elbow* (Moriarty *et al.*, 2009 ▸), *PRODRG* (Schüttelkopf & van Aalten, 2004 ▸), and *pyrogen* in *Coot*.

Additionally, graphical editors can be used to create new or edit existing ligands. There are many ligand editors available; those available as part of the *CCP*4 suite include *JLigand* (Lebedev *et al.*, 2012 ▸) and *Lidia* (Emsley, 2017 ▸), both of which are closely integrated with *Coot*. *JLigand* is a three-dimensional sketcher, which notably allows the creation and modification of links, as well as three-dimensional structure regularization. *Lidia* (the ligand builder in *Coot*) is a two-dimensional sketcher, which notably allows the user to perform an Sbase search (Pongor *et al.*, 1993 ▸) to enable a search for similar ligands.

**Figure 2:**
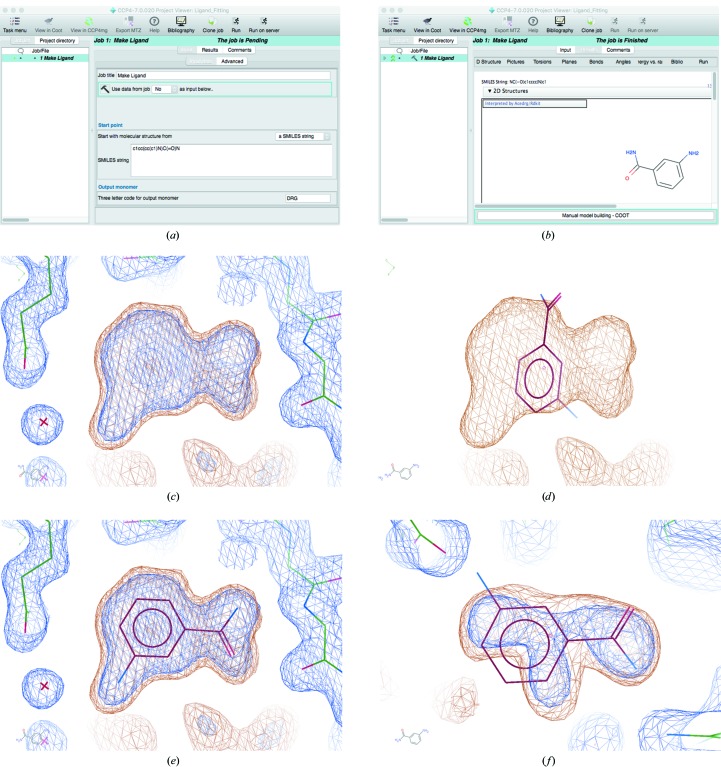
Automatic ligand fitting in *CCP*4. The SMILES string corresponding to 3-aminobenzamide is pasted into the ‘Make Ligand’ task interface in *CCP*4*i*2 (*a*). Upon running the job, *AceDRG* is used to generate ligand restraints and *RDKit* is used to generate an initial conformer and a two-dimensional representation of the ligand (*b*). The ‘Manual Model Building’ task is then executed to open *Coot*. (*c*) displays the model (sticks) and 2*mF*_o_ − *DF*_c_ density map (blue) corresponding to a structure solved with data extending to 2 Å resolution (PDB entry 3kcz; Karlberg *et al.*, 2010 ▸) after manually removing the ligands. The maps are shown using *Coot*’s default contour levels. Automatic ligand fitting is performed, using 3-aminobenzamide as the target. The focal region corresponds to the top-ranked blob identified in the masked map (orange). The ligand coordinates are nominally positioned onto the centre of the blob (*d*). The ligand is then optimally oriented and rigid-body refined into the masked density (*e*). In this case, manual intervention would be required in order to ensure favourable hydrogen bonds are satisfied: this issue is further addressed in §[Sec sec6]6 and Fig. 7 ▸. Multiple blobs are found in the map and ligands are fitted into them; the third-highest ranked ligand is automatically fitted into a blob that actually corresponds to a glycerol molecule (*f*).

The ‘Make Ligand’ task in the *CCP*4*i*2 GUI allows a ligand to be created/imported from a SMILES string or MOL file, or manually created using the *Lidia* sketcher. For example, in Fig. 2 ▸(*a*) the SMILES string corresponding to 3-aminobenzamide is shown pasted into the interface. Upon running the job, *AceDRG* is used to generate ligand restraints and *RDKit* is used to generate an initial conformer and a two-dimensional representation of the ligand (Fig. 2 ▸ *b*). This task can be directly followed by the ‘Manual Model Building’ task, which allows the ligand model and description to be loaded into *Coot* ready for subsequent ligand fitting and refinement.

## 5. Ligand fitting with *Coot*   

Following generation of the ligand description and initial coordinates, the next step is ligand fitting. This is typically performed in real space, and involves attempting to correctly position and orient the ligand as well as selecting the correct conformation. The following sections describe some of the tools available in *Coot* for real-space fitting, including tools for making post-translational modifications and carbohydrate fitting. Details of the tools implemented in *Coot* for dealing with ligands have been described by Debreczeni & Emsley (2012 ▸).

### 5.1. Finding the ligand position and an initial pose   

*Coot* allows an exhaustive search of the map to be performed in order to find all unmodelled blobs that may correspond to potential ligands.

Finding the ligand position involves the following fully automated procedure.

**Figure 3:**
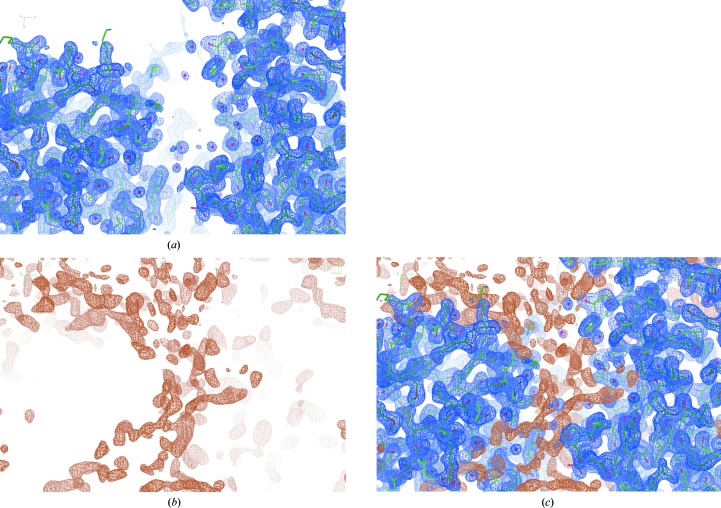
Creation of a mask (PDB entry 3kcz; Karlberg *et al.*, 2010 ▸). The 2*mF*_o_ − *DF*_c_ electron-density map corresponding to the current model is coloured blue (*a*). The mask, coloured orange, is created by artificially setting all modelled regions of the map to zero (*b*). Both maps are shown using the default contour levels in *Coot*. The mask is (by default) shown at a lower contour level (0.2) than the original map (0.55), emphasizing the ‘noise’ in the unmodelled regions and the fact that the masked map is set to zero in regions that have already been modelled. For reference, both masks are also shown together (*c*).

(i) Mask the map. The mask is around the (for example protein) coordinates and is used to set all regions of the density map that have already been modelled to zero (for details, see Emsley & Cowtan, 2004 ▸). This results in a map in which all nonzero density corresponds to unmodelled regions (see Fig. 3 ▸). Note that it can be specified whether or not modelled waters are to be treated as part of the model during mask calculation.
(ii) Find blobs. The masked map is searched for very dense regions. Specifically, blobs of density above a certain threshold are identified, which correspond to unmodelled regions where there is a substantial amount of density. These blobs should be sufficiently sizeable and dense to suggest that a modellable part of the model is missing, and not just correspond to noise (*e.g.* as is often found in solvent regions). Consequently, the blobs represent potential sites where ligands may be located.
(iii) Cut the blobs out.
(iv) Rank the blobs according to density volume.
(v) Fit the target ligand(s) into each of the identified blobs using the following protocol.
   (1) Match centres.
   (2) Orient optimally, hopefully resulting in a sensible orientation (this is performed by matching eigenvalues and eigenvectors).
   (3) Rigid-body refine, so as to quickly optimize the fit to density.
(vi) Finally, score the ligand–blob matches according to the fit of the ligand to the blob (density correlation).

The result is an array of ligands fitted into multiple blobs of density in the map. In some cases false positives will be returned as hits; these need to be manually rejected. Ideally, the agreement between ligand and blob would in some cases be sufficient to warrant continuing with further fitting and refinement. Such acceptable ligands need to be merged into the working model before continuing with the ligand-fitting process (using the ‘Merge Molecules’ tool in the ‘Calculate’ menu).

Fig. 2 ▸ shows an example of automatic ligand fitting. Following ligand description creation, automatic ligand fitting is performed in *Coot* using the ‘Find Ligands’ tool (which is found in the ‘Calculate’ menu under ‘Other Modelling Tools’). The whole masked map is searched in order to identify blobs exhibiting the highest volume of density above a given threshold (Fig. 2 ▸ *c*). The blobs are enumerated and the target ligand is automatically fitted into each of the blobs, optimizing the agreement between model and density (Fig. 2 ▸ *e*). The blobs are then ranked according to the density correlation score. In this case, the agreement between model and density for the top two ranked ligands is compelling. However, the fit for the third-ranked blob appears to be far less convincing (Fig. 2 ▸ *f*); this blob actually corresponds to a glycerol molecule. Indeed, manual inspection of results is required in order to decide which fitted ligands are reasonable enough to carry through to the model for further refinement.

### 5.2. Conformer generation   

Ligand fitting is more complicated for larger ligands that may adopt different conformations depending on their structural environment. By default, the search model used by *Coot* is the ‘low-energy’ conformer output by, for example, *AceDRG* (*via RDKit* and *REFMAC*5). This single conformer is generated with no reference to the electron density, so there is no guarantee that the ligand conformation corresponds to that in the crystal structure. However, *Coot* can perform conformer generation as part of the ligand-fitting process. Specifically, ligands with rotatable bonds can be sampled in many conformations, so that there is a better chance of finding one that fits the density well.

In *Coot*, conformer generation follows the following procedure.

(i) Generate torsion angles. For each rotatable bond, torsion angles are randomly sampled according to the distributions specified in the ligand dictionary, so as to ensure that only models with sensible torsion angles are trialled. Torsions marked as ‘const’, those involving H atoms and those within rings are excluded from consideration.
(ii) Generate conformers. Rotatable bonds are rotated according to the torsion angles generated in the previous step, resulting in sets of initial coordinates corresponding to the conformers. The number of conformers to trial can be adjusted (the default is 50).
(iii) Optimize coordinates. Since the initial conformers may include clashes, the ligand geometry is idealized in order to avoid high-energy conformations.

Each of the resultant conformers is then rigid-body fitted into the density as before (see §[Sec sec5.1]5.1). The ligand conformation with the best density correlation is returned as the result. Fragmenting is recommended for very large ligands with a large number of rotatable bonds; this involves splitting the ligand into multiple parts, fitting the ‘core’ of the ligand using the procedure outlined above and then extending the model outwards from this core into the density (Terwilliger *et al.*, 2006 ▸).

**Figure 4:**
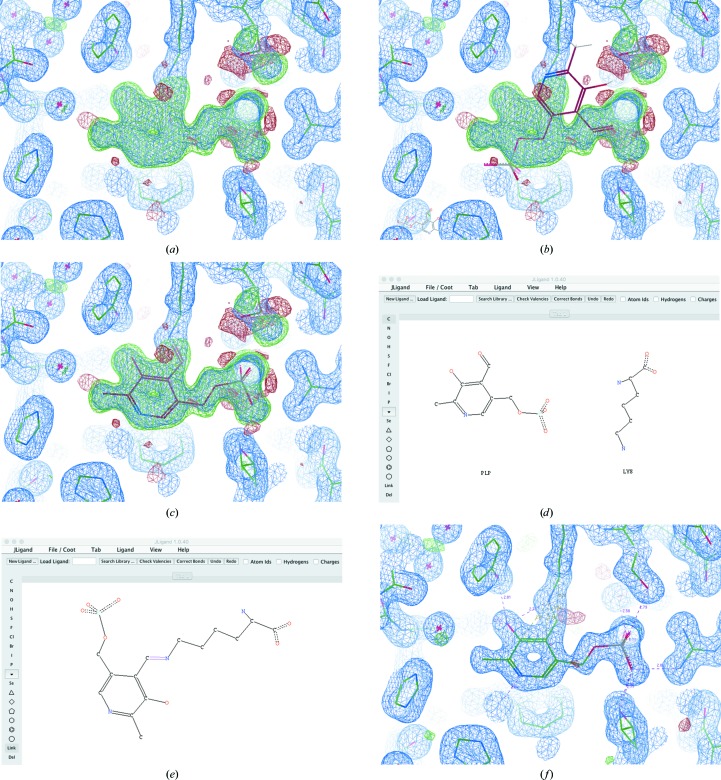
Conformer generation, ligand fitting and link creation, exemplified using pyridoxal 5′-phosphate (monomer code: PLP). (*a*) displays an unmodelled blob in the density of a structure solved using data extending to 1.6 Å resolution (PDB entry 1ajs; Rhee *et al.*, 1997 ▸) after manually removing the ligand from the deposited model. When importing the ligand, the model will not necessarily be in the correct pose or conformation (*b*). Automatic conformer generation and ligand fitting results in a more reasonable pose and conformation for the coenzyme (*c*). Creation of a link record, describing the bond between the N atom in the side chain of Lys258 and a C atom in PLP, can be achieved by opening the Lys and PLP in *JLigand* (*d*). The O atom (O4A) in PLP can then be removed and the double-bond link created between the lysine N atom (NZ) and the C atom (C4A) in PLP (*e*). The model is shown following subsequent refinement by *REFMAC*5 (*f*).

Fig. 4 ▸ demonstrates the conformer generation and ligand fitting of pyridoxal 5′-phosphate (PLP). This ligand has four rotatable bonds, so conformer generation will help to maximize the chance of ligand-fitting success. The unmodelled blob (Fig. 4 ▸ *a*) can easily be identified using the ‘Difference Map Peak Analysis’ tool in *Coot* (which is found in the ‘Validate’ menu). Initial coordinates can be imported by typing ‘PLP’ into the ‘Get Monomer’ tool (found in the ‘File’ menu). However, the imported ligand will not necessarily be in the correct conformation (Fig. 4 ▸ *b*). Conformer generation and ligand fitting can be performed using the ‘Find Ligands’ tool (which is found in the ‘Calculate’ menu under ‘Other Modelling Tools’), resulting in a more reasonable binding mode and conformation for the coenzyme (Fig. 4 ▸ *c*). The new ligand can then be merged into the model using the ‘Merge Molecules’ tool (found in the ‘Calculate’ menu). However, at this point the ligand may still contain errors: real-space refinement and manual intervention may be required in order to resolve any issues. For example, it is clear that the side chain of Ser257 requires attention (Fig. 4 ▸ *c*, upper right quadrant).

### 5.3. Post-translational modifications   

Post-translational modifications (for example, glycosyl­ations, phosphorylations, methylations *etc.*) are dealt with using link records, which are used to specify that there is a particular chemical interaction between a given atom pair. These records are not used to specify restraint targets; they simply state that there is a bonding interaction. Note that they are only needed for nonstandard bonds. For example, link records are not required for peptide bonds or for the bonds within an amino acid; these are implicit and the corresponding restraints in the *CCP*4/*REFMAC* monomer library are automatically used. Also, link records are not used to specify the restraints within a given compound; such restraints would typically be specified in a CIF file specific to the compound (if not already present in the monomer library). Rather, link records are used to specify bonding interactions that are a result of post-translation modifications, either between or within molecules.

**Figure 5:**
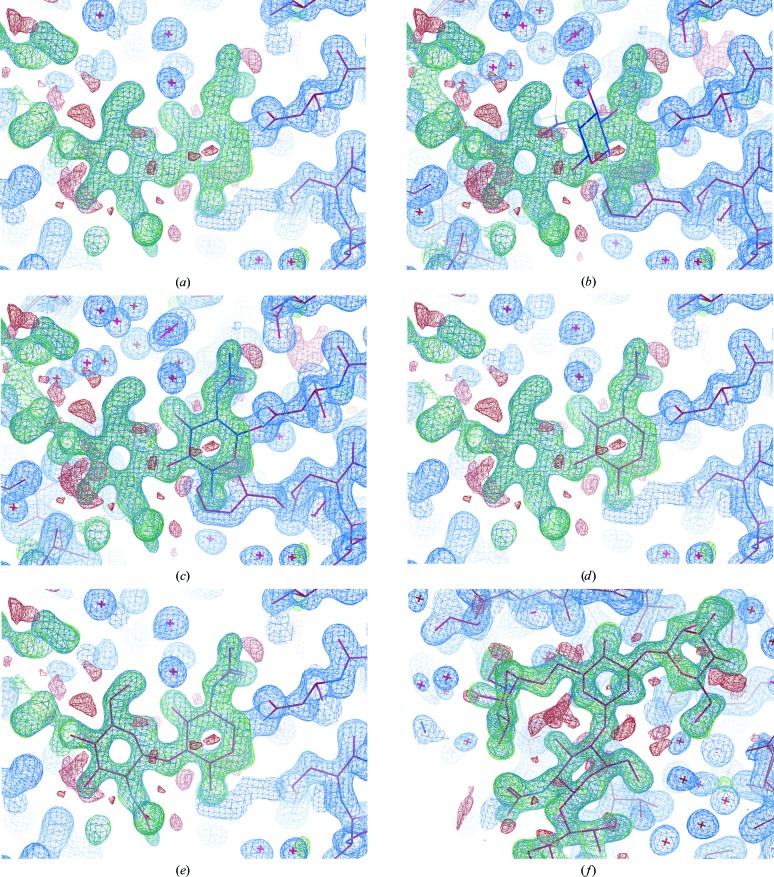
Semi-automatically building an oligosaccharide using *LO*/*Carb*. (*a*) focuses on the glycosylation site of a structure refined using data extending to 1.6 Å resolution (PDB entry 4gos; Jeon *et al.*, 2014 ▸), after removing the carbohydrate. The current model is shown (sticks) along with the 2*mF*_o_ − *DF*_c_ density map (blue) and difference density map (green/red). The positive difference density corresponds to the missing carbohydrate structure, which we know comprises five sugars: two *N*-acetyl-d-glucosamines (NAG), one β-d-mannose (BMA) and two α-d-mannoses (MAN). The carbohydrate is N-linked to the protein residue Asn112. The carbohydrate structure is built by first placing a NAG conformer into the density next to Asn112 (*b*) using the ‘Get Monomer’ tool in *Coot* (found in the ‘File’ menu). After deleting the H atoms, Jiggle Fit (hotkey ‘J’) is used to quickly position and orient the sugar correctly, before manual adjustment and real-space refinement (hotkey ‘r’) to fit the model into the density (*c*). The O atom which visibly clashes with an N atom in Asn112 can then be removed from the glycosylation site (*d*), and the new sugar merged into the existing model (using the ‘Merge Molecules’ tool found in the ‘Calculate’ menu). *LO*/*Carb* functionality is accessible *via* the ‘Glyco’ menu (which is activated by selecting ‘Carbohydrate’ from the ‘Modules’ section in the ‘Extensions’ menu), through which it is possible to automatically add and fit the additional NAG (*e*) and finally the remaining BMA and two MAN sugars (*f*). Note that in this case *REFMAC*5 will automatically create the N-link record when the model is next refined, owing to it being a standard link present in the monomer library.

The *CCP*4/*REFMAC* monomer library contains knowledge of many standard post-translational modifications. Consequently, in many cases standard links are created (by *REFMAC*5) based on proximity detection. The restraints corresponding to such links are applied automatically during refinement by *REFMAC*5, and the link records are added to the output PDB file (as in Fig. 5 ▸).

Link records for nonstandard post-translational modifications need to be created manually, for example using *JLigand*. Once created, the link record (for the PDB file) can be exported, along with the CIF file describing the restraint. The integrated solution linking *CCP*4*i*2, *Coot* and *JLigand* allows links to be created, applied and transferred automatically.

For example, in Fig. 4 ▸ a link record needs to be created to describe the bond between the N atom in the side chain of Lys258 and a C atom in PLP. Additionally, an O atom in PLP, which is replaced by the lysine N atom, needs to be removed. This can be achieved using *JLigand* (Fig. 4 ▸ *d*), which can be launched from within *Coot via* the ‘Modelling’ section of the ‘Extensions’ menu. The O atom (O4A) in PLP can be removed and the double-bond link created between the lysine N atom (NZ) and the C atom (C4A) in PLP (Fig. 4 ▸ *e*). Owing to software integration, the link record is automatically transferred back to the model in *Coot*, and in turn back to the *CCP*4*i*2 project. The model-building task can then be followed up by full-model refinement with *REFMAC5*, before opening the results in *Coot* for further inspection. The resultant model looks reasonable (Fig. 4 ▸ *f*), with the link correctly utilized (displayed as a dashed line). Note also the hydrogen-bonding network, which includes the side chain of Ser257 that is now stabilized in the correct conformation owing to the interaction with PLP (upper right quadrant).

### 5.4. Jiggle Fit   

Jiggle Fit is used to optimize the fit of the current model to density, using a combination of trialling positions and orientations, rigid-body fitting and real-space refinement. Using Jiggle Fit, it is possible to start from a conformer that is roughly placed near the correct location, the outcome being a well fitted model ready for manual attention (see Fig. 5 ▸).

The Jiggle Fit algorithm may be described as follows.

(i) Randomly sample small transformations (translations and rotations).
(ii) For each transformation:
   (1) transform the model coordinates accordingly;
   (2) rigid-body fit to the density;
   (3) score the fit to density (density correlation).
(iii) Real-space refine the best-scoring pose.

Jiggle Fit (hotkey ‘J’) complements the real-space refine (hotkey ‘r’) and sphere refine (hotkey ‘R’) tools. Sphere refine performs real-space refinement on the target residue/molecule/region as well as on the surrounding environment within a certain radius, for example allowing the co-optimization of a ligand and its environment (for details, see Emsley *et al.*, 2010 ▸).

Note that Jiggle Fit has a very wide radius of convergence, in contrast to real-space refinement. Indeed, Jiggle Fit has proven to be useful not only for ligands but also for larger regions (protein domains, nucleic acids *etc.*), including the fitting of macromolecular domains into cryo-EM reconstructions (Brown *et al.*, 2015 ▸).

### 5.5. Carbohydrate building   

It has been acknowledged that a high proportion of the models in the PDB contain significant errors in carbohydrate stereochemistry (Agirre, Davies *et al.*, 2015 ▸; Crispin *et al.*, 2007 ▸). This emphasizes the lack of, and the necessity for, improved carbohydrate-specific building and validation tools. As a consequence, substantial advancements have recently been made in the carbohydrate-building capabilities of *Coot*. Notably, the *LO*/*Carb* (*Linking Oligosaccharides/Carbohydrates*) tool has been created with the objective of easing the building of complex carbohydrate structures, allowing them to be quickly and reliably built. This uses a dictionary of standard links, ensuring that reasonable models are built in accordance with prior knowledge regarding the accessible conformational space of glycosidic linkages (Frank *et al.*, 2007 ▸).

For complex poly/oligosaccharide structures, sugars are built one by one, extending the model wherever possible. Starting from a partially built model, *LO*/*Carb* fixes the existing model and attempts to add additional sugars. The fit of the newly built sugar is optimized by trialling various orientations and sampling the space of reasonable torsion angles, followed by local real-space refinement. If the resultant fit to density is not sufficiently good then the sugar is removed. In automatic mode, this procedure is iterated until no more sugars can be built.

**Figure 6:**
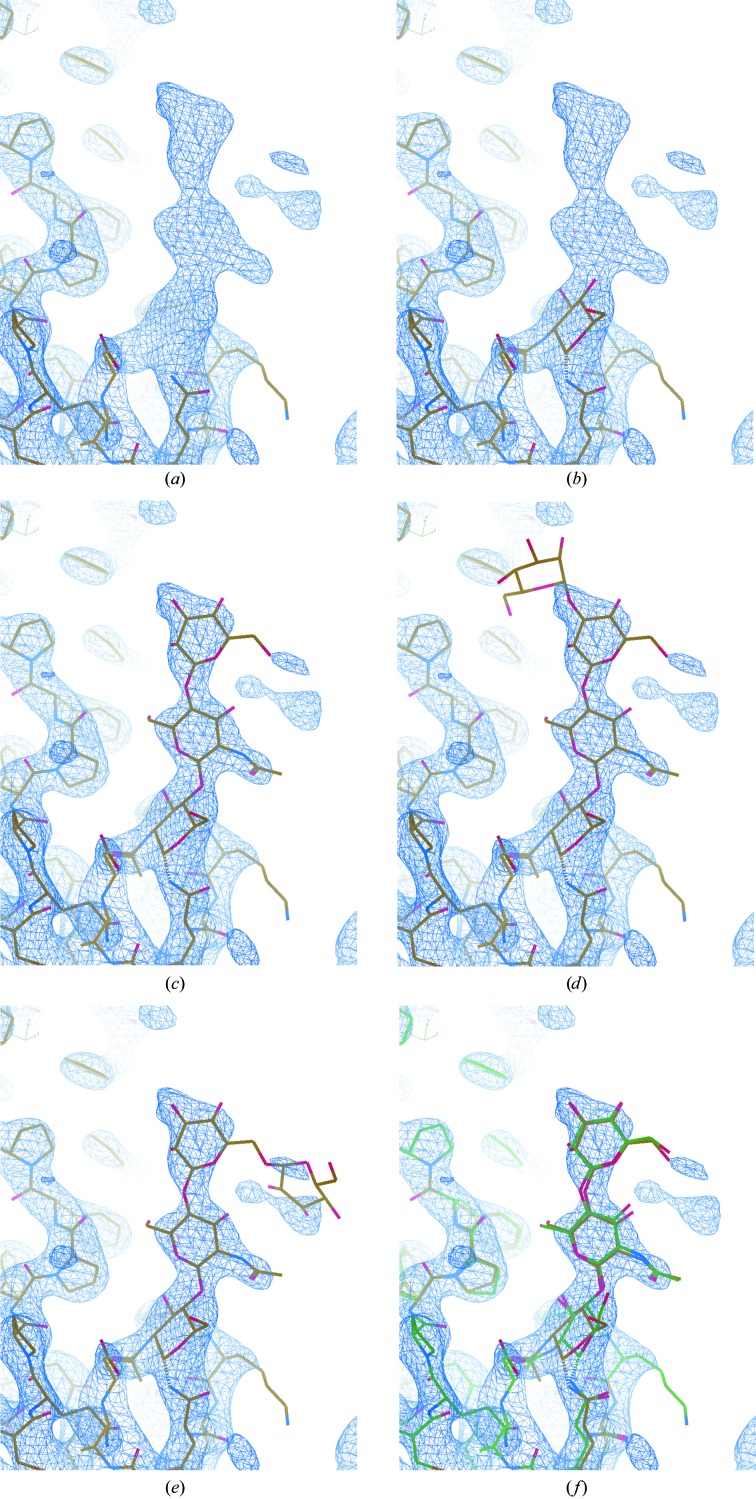
Automatically building an oligosaccharide into a low-resolution map using *LO*/*Carb*. (*a*) focuses on the glycosylation site of a structure refined using data extending to 3.3 Å resolution (PDB entry 4n4z; Gati *et al.*, 2014 ▸) after removing the carbohydrate structure. The current model is shown along with the 2*mF*_o_ − *DF*_c_ density map, using the default contour level in *Coot* (0.34). Selecting ‘Add Oligomannose’ from the ‘Glyco’ menu (which is activated by selecting ‘Carbohydrate’ from the ‘Modules’ section in the ‘Extensions’ menu) results in *Coot* attempting to automatically build as much of the carbohydrate structure as possible. The sugar linked to the protein is built first (*b*), followed by additional sugars one by one, until the whole structure is built (*c*). Attempts to build additional sugars in chemically reasonable positions are made, but are rejected if there is insufficient density to support the model (*d*, *e*). (*f*) shows the final automatically built model (yellow) next to the original deposited model (green).

*LO*/*Carb* functionality can be used in semi-automatic mode, in which the user chooses which particular sugar to build at each step (see Fig. 5 ▸), or in automatic mode, whereby a carbohydrate structure is built without user intervention (see Fig. 6 ▸). These tools can be used over a wide range of resolutions (see Fig. 6 ▸).

## 6. Full-model refinement, analysis, validation and visualization   

Following successful ligand building and fitting, full-model refinement can then be performed. This allows the ligand and protein to co-refine, synergistically optimizing the agreement between model and experimental data. Since the ligand contributes to the model phases, subsequent density interpretation must be performed with care owing to the potential for model bias. Careful inspection of the difference density and OMIT maps is often necessary in order to ensure that the ligand is actually present and in the modelled state.

**Figure 7:**
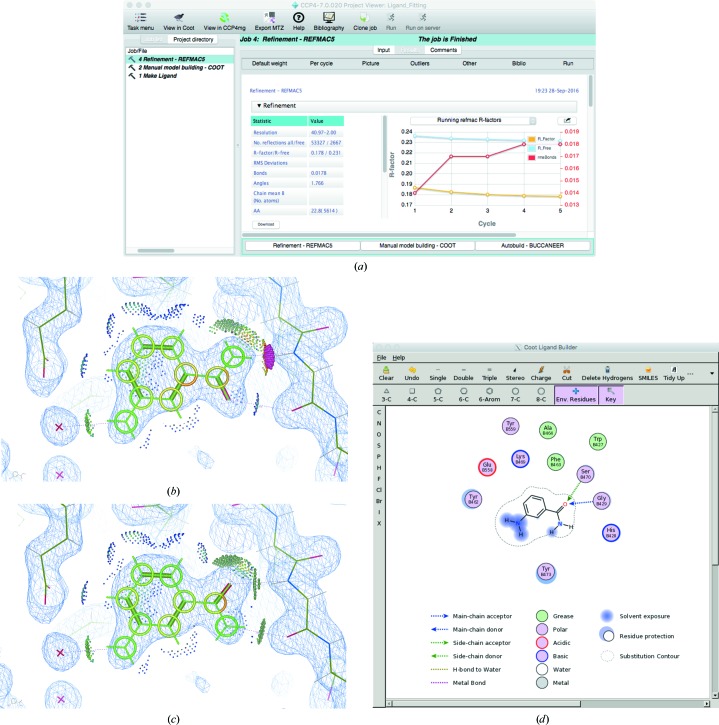
Refinement and validation. (*a*) shows the results of refinement using *REFMAC*5 *via* the *CCP*4*i*2 GUI after fitting 3-aminobenzamide into PDB entry 3kcz as demonstrated in Fig. 2 ▸. After refinement, the ligand is analysed using *Coot* by displaying environment distances, isolated dots and ligand distortions (*b*). It is evident that the ligand is in an incorrect conformation. Swapping the O and N atoms results in better stereochemistry, as is evident after re-refining the model using *REFMAC*5 and re-analysing the model in *Coot* (*c*). Tools such as ‘Torsion General’ are useful for such manual editing (found in the side bar to the right of the main *Coot* window). A two-dimensional depiction of the environment of the ligand is shown using *FLEV* in *Coot* (*d*).

Fig. 7 ▸ builds on the example of ligand fitting shown in Fig. 2 ▸ (in which 3-aminobenzamide is fitted into a density map) by performing subsequent model refinement and validation of the results. The ligand is merged into the original model (using the ‘Merge Molecules’ tool found in the ‘Calculate’ menu), real-space refined (hotkey ‘r’) in order to optimize the fit of the ligand to the density and sphere refined (hotkey ‘R’) in order to co-refine the ligand along with its local environment. The model is then saved to *CCP*4*i*2 (found in the ‘File’ menu). After closing *Coot*, the *CCP*4*i*2 task is followed by full-model refinement using *REFMAC*5 (Fig. 7 ▸ *a*) before reopening *Coot* to display the resultant model and maps.

The ligand is validated by first displaying environment distances (found in the ‘Measures’ menu), which provides information regarding the local hydrogen-bonding network and interactions. Displaying isolated dots (found in the ‘Ligand’ menu) further emphasizes favourable and unfavourable atomic contacts and clashes; this representation is the result of *REDUCE* and *PROBE* being executed (Word, Lovell, LaBean *et al.*, 1999 ▸; Word, Lovell, Richardson *et al.*, 1999 ▸). Displaying ligand distortions (found in the ‘Ligand’ menu) results in all bonds and angles in the ligand being validated by *Mogul* (Bruno *et al.*, 2004 ▸), which uses data derived from the CSD (Groom *et al.*, 2016 ▸). Note that this represents independent cross-validation, as the ligand restraints generated by *AceDRG* are derived from another source: the COD (Gražulis *et al.*, 2009 ▸). Bonds and angles in the ligand are represented as thick lines and arcs, and are coloured according to the *Z*-scores resulting from their comparison against the *Mogul* distributions (red indicates potential problems). These validation features are described in more detail by Emsley (2017 ▸).

In this case, consideration of the local environment of the ligand reveals the ligand to be in an incorrect conformation owing to unfavourable contacts and interactions (Fig. 7 ▸ *b*). Swapping the O and N atoms in the ligand results in more sensible stereochemistry, and thus the correct conformation (Fig. 7 ▸ *c*). Repeating the ligand-validation analysis in the correct conformation provides visual feedback indicating that the unfavourable contacts have been resolved and also that some of the worst angles now adopt more reasonable values. Opening *Flatland Ligand Environment View* (*FLEV*; Emsley, 2017 ▸) in *Lidia* (found in the ‘Ligand’ menu of *Coot*) provides a two-dimensional depiction of the local environment of the ligand, emphasizing important interactions, contacts and chemical features (Fig. 7 ▸ *d*).

Note that looking at the *B* factors of the O and N atoms would also have indicated that they should be swapped: in the incorrect conformation the *B* factors of the O and N atoms are 17.7 and 12.5 Å^2^, respectively, whereas in the correct conformation the *B* factors are 13.8 and 15.2 Å^2^ (after full-model refinement). In the absence of strong evidence to suggest otherwise, proximal atoms would always be expected to exhibit similar *B* factors. Consequently, this information would lead us to select the correct model: a model exhibiting nearby atoms with *B* factors of 13.8 and 15.2 Å^2^ is more reasonable than one with *B* factors of 17.7 and 12.5 Å^2^. Indeed, the analysis of *B* factors can provide useful information when assessing potential model correctness. Note that it is the relative difference between (and distribution of) *B* factors within the same model that provides useful information; the absolute values of *B* factors cannot be meaningfully compared between different models.

Another feature that can help one to gain intuition is map blurring (found under ‘Map Sharpening’ in the ‘Calculate’ menu in *Coot*), which involves adding a very high *B* factor to the density map in order to give higher weight to the lower resolution reflections. This can provide evidence for the presence of structure in the crystal that is currently missing from the model, especially in cases where the ligand is particularly flexible. Indeed, viewing different types of density maps can facilitate the extraction of as much information as possible.

Finally, validation is also performed by the wwPDB (Berman *et al.*, 2003 ▸) prior to model deposition. The wwPDB validation report provides an assessment of structure quality using a variety of criteria. Attempts have been made to improve PDB validation (Read *et al.*, 2011 ▸), and notably this report now includes the local ligand density fit (LLDF), which compares the density correlation of the ligand with that of the surrounding modelled regions. Note also that *Privateer* (Agirre, Iglesias-Fernández *et al.*, 2015 ▸; Agirre, 2017 ▸) can be used for the conformational validation of carbohydrate structures. Such tools can aid the assessment of model quality when approaching model completion.

## 7. Discussion   

In this paper, some of the features available in various software tools designed to ease ligand building, fitting, refinement and validation have been described, and a number of scenarios exemplifying practical usage have been considered. These tools, distributed as part of the *CCP*4 suite, include *AceDRG* for the generation of ligand restraint dictionaries and conformers, *Coot* for map interpretation, ligand finding, fitting, conformer generation, real-space refinement, Jiggle Fit and carbohydrate building (*LO*/*Carb*), *JLigand* for manually creating and editing ligands, restraints and link records in three dimensions, *Lidia* for manually creating and editing ligands in two dimensions, as well as visualization and analysis of the chemical environments of molecules (*FLEV*), and *REFMAC*5 for macromolecular refinement. Along with the *CCP*4*i*2 GUI, these tools aim to ease the ligand-fitting process, facilitating the ability for ligands to be modelled quickly and reliably whilst providing diagnostics to help tackle the varied problems that may be encountered during the procedure. Substantial advancements have recently been made, designed to make life easier for the computational crystallographer. However, ultimately, there is no substitute for manual inspection and due diligence.

The issues encountered during ligand modelling may vary: at higher resolutions multiple conformations, partial occupancies and identifying the correct protonation/tautomeric state can prove a challenge, whereas at lower resolutions the problems encountered usually relate to density interpretation. For example, ligands may only be partially visible, and thus only part of the ligand can modelled. This could be owing to the ligand or active site being particularly flexible. Alternatively, it could be that the ligand has been truncated/modified, or that the blob in question corresponds to a completely different compound.

Problems affecting the ability to successfully model a ligand can be caused by a variety of factors, including the following.

(i) Poor data quality and/or low resolution. If possible, collecting data of better quality is always the best solution; poor-quality data cannot be fixed/improved by downstream processing.
(ii) Incorrect/suboptimal data processing, for example an incorrect space group, which may have the effect of reducing effective data quality or the ability to appropriately model the crystal contents. Soaking a crystal can change its space group.
(iii) Radiation damage, which accumulates, causing the crystal contents to change throughout data collection. This essentially means that observations on different images correspond to different crystal structures. In some cases this simply causes an effective reduction of resolution, but in others the affects of radiation damage can be more targeted/localized, creating the impression of multiple conformations.
(iv) An insufficiently refined model, and thus inaccurate phases. Since changing one part of the model has an effect on the whole density map, the appearance of binding-site density can dramatically change (and ideally become more reliable) as other parts of the model are built and refined.
(v) Incorrect modelling in the active site, which can cause model bias. It is always important to question the reliability of the density; this is particularly important at medium and lower resolutions.

Indeed, it is important for the rest of the model to be built and refined as well as possible before even thinking about looking at potential ligands or binding sites (despite the temptation). Modelling in the binding sites should be delayed until the last possible moment, including the addition of waters, except perhaps where evidence is incontrovertible. Incorrect building causes model bias, particularly at lower resolutions, and misleading density ultimately leads to a poor-quality (and possibly grossly inaccurate) model, which may result in incorrect biological conclusions. It is also important to ensure that not only does the model reasonably fit into the density, but that it makes chemical sense. This can be complicated and challenging, requiring a sufficient degree of knowledge of chemistry (Bax *et al.*, 2017 ▸). The conclusion is: take care.

It is also important to check the crystallization conditions and buffer composition in order to gain intuition regarding what compounds might be present in the crystal structure. For example, an unconvincingly fitted ligand built into a binding site might actually turn out to be a glycerol molecule (as in Fig. 2 ▸).

The models of protein–ligand complexes are often a result of subjective interpretation. In some cases, there may be a tendency for the over-interpretation of density (wishful thinking). However, it is always better to be conservative, critical and honest.
